# The structure of the AliC GH13 α-amylase from *Alicyclobacillus* sp. reveals the accommodation of starch branching points in the α-amylase family

**DOI:** 10.1107/S2059798318014900

**Published:** 2019-01-04

**Authors:** Jon Agirre, Olga Moroz, Sebastian Meier, Jesper Brask, Astrid Munch, Tine Hoff, Carsten Andersen, Keith S. Wilson, Gideon J. Davies

**Affiliations:** a York Structural Biology Laboratory, Department of Chemistry, The University of York, York YO10 5DD, England; b Department of Chemistry, Technical University of Denmark, 2800 Lyngby, Denmark; c Novozymes A/S, Krogshoejvej 36, 2880 Bagsvaerd, Denmark

**Keywords:** AliC GH13 α-amylase, starch branching points, glycoside hydrolases, pullulan, carbohydrate-active enzymes, *Alicyclobacillus*

## Abstract

α-Amylases are glycoside hydrolases that break the α-1,4 bonds in starch and related glycans. The degradation of starch is rendered difficult by the presence of varying degrees of α-1,6 branch points and their possible accommodation within the active centre of α-amylase enzymes. Given the myriad industrial uses for starch and thus also for α-amylase-catalysed starch degradation and modification, there is considerable interest in how different α-amylases might accommodate these branches, thus impacting on the potential processing of highly branched post-hydrolysis remnants (known as limit dextrins) and societal applications. Here, it was sought to probe the branch-point accommodation of the *Alicyclobacillus* sp. CAZy family GH13 α-amylase AliC, prompted by the observation of a molecule of glucose in a position that may represent a branch point in an acarbose complex solved at 2.1 Å resolution. Limit digest analysis by two-dimensional NMR using both pullulan (a regular linear polysaccharide of α-1,4, α-1,4, α-1,6 repeating trisaccharides) and amylopectin starch showed how the *Alicyclo­bacillus* sp. enzyme could accept α-1,6 branches in at least the −2, +1 and +2 subsites, consistent with the three-dimensional structures with glucosyl moieties in the +1 and +2 subsites and the solvent-exposure of the −2 subsite 6-hydroxyl group. Together, the work provides a rare insight into branch-point acceptance in these industrial catalysts.

## Abstract 2

In the light of NMR data on product profiles, the structure of an *Alicyclobacillus* sp. CAZy family GH13 α-amylase highlights the accommodation of branch points in the α-amylase active centre.

## 1. Introduction   

The enzymatic hydrolysis of starch is not merely central to human health and nutrition, but also to a vast and diverse array of industries. Starch degradation is central to the production of high-fructose corn syrups, modern detergents and starch-derived biofuels, in brewing and fermentation, and in the adhesive, textile and paper sectors. The estimated value of the starch market in 2018 has been proposed to be around $77 billion, growing at a rate of greater than 7% per year (http://www.prweb.com/pdfdownload/10923341.pdf; de Souza & de Oliveira e Magalhães, 2010 ▸). There is thus a massive interest in the enzymatic degradation and modification of starch from both academic and industrial perspectives (van der Maarel *et al.*, 2002 ▸; Liu & Xu, 2008 ▸). Indeed, the engineering of starch-degrading enzymes, informed by their three-dimensional structure, has been important for their application (reviewed, for example, in Shaw *et al.*, 1999 ▸; Nielsen & Borchert, 2000 ▸). Starch degradation requires a consortium of enzymes, notably endo-acting α-amylases (EC 3.2.1.1) and chain-end-acting glucoamylases (EC 3.2.1.3) in microbes. In recent times these two players have been accompanied by copper-dependent lytic polysaccharide monooxygenases that break down starch, including highly recalcitrant forms, through an oxidative mechanism (Vu *et al.*, 2014 ▸; Lo Leggio *et al.*, 2015 ▸).

The majority of endo-acting α-amylases in industrial starch-degradation processes are CAZy (http://www.cazy.org; see Lombard *et al.*, 2014 ▸) family GH13 enzymes. GH13 is one of the most well studied glycoside hydrolase families (reviewed in CAZypedia at http://www.cazypedia.org/index.php/Glycoside_Hydrolase_Family_13; The CAZypedia Consortium, 2018 ▸). Over 111 different three-dimensional structures of GH13 enzymes are now known (see http://www.cazy.org/GH13_structure.html). One particularly important subset of GH13 enzymes are the ‘Termamyl’-like α-amylases, historically named after an enzyme from *Bacillus licheniformis*. These enzymes typically feature a three-domain ‘A, B, C’ arrangement with a C-terminal β-sheet domain and with domain B being a protrusion from the (β/α)_8_ fold of domain A. The catalytic centre is placed in domain A, whilst the A–B interface forms the substrate-binding cleft. Many three-dimensional structures of ‘Termamyl’-like α-amylases are known. Some notable members include that from *B. licheniformis* (Machius *et al.*, 1995 ▸), a chimeric *B. licheniformis*/*B. amyloliquefaciens* enzyme (Brzozowski *et al.*, 2000 ▸), an enzyme from *Geobacillus stearothermophilus* (Suvd *et al.*, 2001 ▸) and an enzyme from *B. halmapalus* (Davies *et al.*, 2005 ▸). Notably, as well as having stabilizing Ca^2+^ ions in various domains, a characteristic Ca^2+^–Na^+^–Ca^2+^ triad is observed at the A/B-domain interface (for the historical context, see Machius *et al.*, 1998 ▸; Brzozowski *et al.*, 2000 ▸).

Currently, the CAZy classification lists over 100 different three-dimensional structures of α-amylases from family GH13. Remarkably, to our knowledge only one of these, the *Bacteroides thetaiotaomicron* SusG protein, contains a branched substrate within its active centre. In this case, following elegant work by the Koropatkin and Brumer groups (Arnal *et al.*, 2018 ▸), an α-1,6 branch was observed in the +1 subsite. The GlgE protein from *Streptomyces coelicolor* (PDB entry 5lgw; Syson *et al.*, 2016 ▸) also contains a branched oligosaccharide, but this ligand is bound far from the active centre and is instead located on a distal starch-binding domain. Here, we report the three-dimensional structure of a ‘Termamyl’-like α-amylase, the AliC α-amylase from *Alicyclobacillus* sp. 18711. An initial ligand-bound structure with a transglycosyl­ated acarbose-derived oligosaccharide at a resolution of 2.1 Å revealed a noncovalently linked glucose moiety, hinting at a putative branch-accommodation site around the +2/+3 subsites. A subsequent lower resolution (approximately 3 Å) analysis revealed the binding of a branched ligand in the +1/+2 subsites with an α-1,6-linked glucose branch bound to the +1 subsite sugar. Motivated by these observations, two-dimensional NMR was used to map the subsite branch preferences on the basis of the structures of the observed limit dextrin products, highlighting how the AliC α-amylase can accommodate amylopectin and pullulan substrates.

## 2. Methods   

### 2.1. Crystallization   

*Alicyclobacillus* sp. 18711 α-amylase (GenBank MH533021) was a kind gift from Novozymes A/S (Bagsvaerd, Denmark), where it had been cloned in a strain variant of *B. subtilis* PL1801 from *Alicyclobacillus* sp. 18711 isolated from a Danish forest floor. A two-amino-acid deletion (T182*G183*) was introduced by SOE PCR (Higuchi *et al.*, 1988 ▸) using synthetic oligonucleotides purchased from Invitrogen, and the α-amylase variant was expressed by fermenting at 37°C for four days in a soy- and starch-based broth.

The fermentation supernatant was filtrated through a 0.45 µm filter followed by filtration through a 0.2 µm filter. After the addition of 1 *M* ammonium sulfate and adjustment of the pH to pH 8, the supernatant was applied onto a 69 ml Butyl TOYOPEARL column. Prior to loading, the column had been equilibrated in three column volumes (CV) of 25 m*M* borate pH 8, 2 m*M* CaCl_2_, 1 *M* ammonium sulfate. In order to remove unbound material, the column was washed with 3 CV of 25 m*M* borate pH 8, 2 m*M* CaCl_2_, 1 *M* ammonium sulfate. Elution of the target protein was obtained with a decreasing salt gradient from 1 to 0 *M* ammonium sulfate in 25 m*M* borate pH 8, 2 m*M* CaCl_2_, followed by 3 CV of 100% 25 m*M* borate pH 8, 2 m*M* CaCl_2_. The flow rate was 10 ml min^−1^. Relevant fractions were selected and pooled based on the chromatogram and on SDS–PAGE analysis. The amylase activity of the purified enzymes was confirmed using the AMYL liquid amylase assay (Roche/Hitachi system).

#### 2.1.1. Acarbose complex   

For the acarbose complex, co-crystallization screening was carried out using sitting-drop vapour diffusion with drops set up using a Mosquito Crystal liquid-handling robot (TTP LabTech, UK) with 150 nl protein solution plus 150 nl reservoir solution in 96-well format plates (MRC 2-well crystallization microplates; Swissci, Switzerland) equilibrated against 54 µl reservoir solution. Initial crystallization experiments were carried out at room temperature using a number of commercial screens. Diffraction-quality crystals were obtained in PACT screen condition G11 [0.2 *M* sodium citrate, 0.1 *M* bis-tris propane (BTP) pH 6.5, 20% PEG 3350]. The crystals were tested in-house prior to being sent to the synchrotron. Crystallization conditions are given in Table 1 ▸.

#### 2.1.2. Branched-ligand complex   

Crystals of the complex with 20 m*M* 6^3^-α-d-glucosyl-maltotriose (GMT; a branched ligand) were obtained by manual optimization in a 24-well Linbro tray (hanging drops) in 20% PEG 3350, 0.1 *M* BTP pH 8.5, 0.2% sodium sulfate with seeding. The initial seeding stock was prepared by crushing crystals of the acarbose complex, adding 50 µl mother liquor and vortexing the mixture for 1 min using a Seed Bead (Hampton Research), based on the protocol described in D’Arcy *et al.* (2014 ▸). Different seed dilutions were screened; the final crystals grew using a 1:1000 seed dilution.

#### 

Details of the crystallization experiments are given in Table 1 ▸.

### 2.2. Data collection and processing, structure solution and refinement   

Computations were carried out using programs from the *CCP*4 suite (Winn *et al.*, 2011 ▸) unless otherwise stated. For the structure of the acarbose complex, data were collected to 2.1 Å resolution on beamline I04 at Diamond Light Source (DLS). The crystal belonged to space group *P*4_1_2_1_2, with unit-cell parameters *a* = *b* = 180.90, *c* = 77.85 Å. The data were processed with *xia*2 (Winter *et al.*, 2013 ▸). The structure was solved using *MOLREP* (Vagin & Teplyakov, 2010 ▸) with the maltohexaose-producing amylase from alkalophilic *Bacillus* sp. 707 as a search model (PDB entry 1wp6; Kanai *et al.*, 2004 ▸).

For the branched-ligand complex, data were collected to 2.95 Å resolution on beamline I04 at DLS. The crystals belonged to space group *P*6_1_, with unit-cell parameters *a* = *b* = 212.18, *c* = 172.22 Å. The data were processed with *xia*2 (Winter *et al.*, 2013 ▸). The structure was solved by *MOLREP* using the acarbose complex (minus all ligands) as a search model. Data-collection statistics are given in Table 2 ▸.

Both structures were refined by *REFMAC* (Murshudov *et al.*, 2011 ▸) iterated with manual model correction using *Coot* (Emsley *et al.*, 2010 ▸). Those monosaccharides that were expected to be in their minimal energy conformation (^4^ *C*_1_ for d-glucopyranose) were additionally restrained to adopt torsional values consistent with such a conformation. This was performed using a dictionary containing unimodal dihedral restraints produced by *Privateer* (Agirre *et al.*, 2015 ▸). Including these restraints in the refinement caused the *R*_free_ values to decrease for both structures. The final *R* and *R*_free_ are 0.138 and 0.176 for the acarbose ligand complex and 0.156 and 0.183 for the branched-ligand complex, respectively. Validation was performed using *MolProbity* (Chen *et al.*, 2010 ▸), *EDSTATS* (Tickle, 2012 ▸) and *Privateer* (Agirre *et al.*, 2015 ▸) through the use of the *CCP*4*i*2 interface (Potterton *et al.*, 2018 ▸). During this work, a *MolProbity* graphical interface for *CCP*4*i*2 was developed. Aside from supporting the usual reporting, the functionality of the interface was extended to cover automated 180° rotation of suggested histidine, asparagine and glutamine side chains around the last χ angle, with an additional real-space refinement step, and real-time compression and decompression of the results from *PROBE*, with a typical ratio of reduction in file size of 8:1. This new interface is available in *CCP*4 through use of the ‘Analyse model geometry’ task.

Data-processing and refinement statistics for both structures are given in Table 3 ▸.

### 2.3. Degradation of pullulan and amylopectin by the purified *Alicyclobacillus* α-amylase   

The enzymatic specificity of the *Alicyclobacillus* α-amylase was experimentally determined to complement the search for potential branch-point accommodation in the active site in the crystal structures. To this end, pullulan and amylopectin were subjected to degradation prior to NMR analysis of the fragments formed. Pullulan (Sigma–Aldrich, St Louis, Missouri, USA) samples were degraded by purified *Alicyclobacillus* α-amylase at room temperature and samples were withdrawn, inactivated at 90°C for 10 min, condensed by lyophilization and redissolved in 600 µl D_2_O (99.9%; Cambridge Isotope Laboratories, Andover, Massachusetts, USA) to obtain partially degraded and fully degraded samples. The samples were transferred to 5 mm NMR sample tubes for analysis. Amylopectin (from potato starch; Sigma–Aldrich) was incubated at 30°C overnight with the purified *Alicyclobacillus* α-amylase, inactivated at 90°C for 10 min, condensed by lyophilization and redissolved in 600 µl D_2_O for NMR analysis.

### 2.4. NMR spectroscopy   

All NMR spectra were recorded on an 800 MHz Avance II spectrometer (Bruker, Fällanden, Switzerland) equipped with a TCI Z-gradient CryoProbe and an 18.7 T magnet (Oxford Magnet Technology, Oxford, England). Highly resolved ^1^H–^13^C HSQC spectra employing a sweep width of 10 p.p.m. centred near the ^13^C chemical shift of the α-anomeric signals were recorded as data matrices of 1024 × 256 complex data points sampling acquisition times of 143 and 127 ms in the ^1^H and ^13^C dimensions, respectively. High-precision signal measurements in the two-dimensional spectra were thus used to enumerate the number of signals in the resultant reaction products and for the identification of the products by comparison with authentic standards including glucose, malto­oligosaccharides, panose and limit dextrins (Petersen *et al.*, 2014 ▸, 2015 ▸).

All spectra were processed with extensive zero filling in both dimensions using a shifted sine-bell apodization function and were analysed with *TopSpin* 2.1 pl 5 (Bruker).

## 3. Results and discussion   

### 3.1. Three-dimensional structure of AliC α-amylase and its acarbose-derived complex   

The complex of AliC with acarbose was solved by molecular replacement, with two molecules of AliC in the asymmetric unit, at a resolution of 2.1 Å. The fold, as expected, is a canonical three-domain arrangement with the A, B and C domains defined approximately as A, residues 4–104 and 210–397; B, residues 105–209; and C, residues 398–484. A classical Ca^2+^–Na^+^–Ca^2+^ triad (Machius *et al.*, 1998 ▸; Brzozowski *et al.*, 2000 ▸) is found at the A/B-domain interface. At the time of writing, structural similarity searches using *PDBeFold* (Krissinel & Henrick, 2004 ▸) showed that the closest three-dimensional match to AliC is the *B. halmapalus* α-amylase (Davies *et al.*, 2005 ▸), with 67% sequence identity and with 479 aligned C^α^ atoms overlapping with an r.m.s.d. of 0.49 Å (*PDBeFold Q* score 0.95, *Z*-score 27.8). Other close structural homologs are the malto­hexaose-producing amylase from *Bacillus* sp. 707 (Kanai *et al.*, 2004 ▸) and the calcium-free amylase AmyK38 from *Bacillus* sp. strain KSM-K38 (Nonaka *et al.*, 2003 ▸).

The structure of AliC was determined in the presence of the inhibitor acarbose. As with many (retaining) α-amylase complexes [some examples from the author’s laboratory include those reported in Brzozowski *et al.* (2000 ▸), Davies *et al.* (2005 ▸), Brzozowski & Davies (1997 ▸), Dauter *et al.* (1999 ▸) and Offen *et al.* (2015 ▸)], the acarbose is observed as a transglycosylated species, here a hexasaccharide which contains two of the acarviosin disaccharide motifs. The complex defines six subsites, −4 to +2, with the expected catalytic GH13 signature triad of Asp234 (nucleophile), Glu265 (acid/base) and Asp332 (interacting with O2/O3 of the −1 subsite sugar) all disposed for catalysis, here around the ^2^ *H*_3_ half-chair of the unsaturated cyclohexitol moiety.

### 3.2. Limit digest analysis of the action of AliC on pullulan and amylopectin   

**Figure 1:**
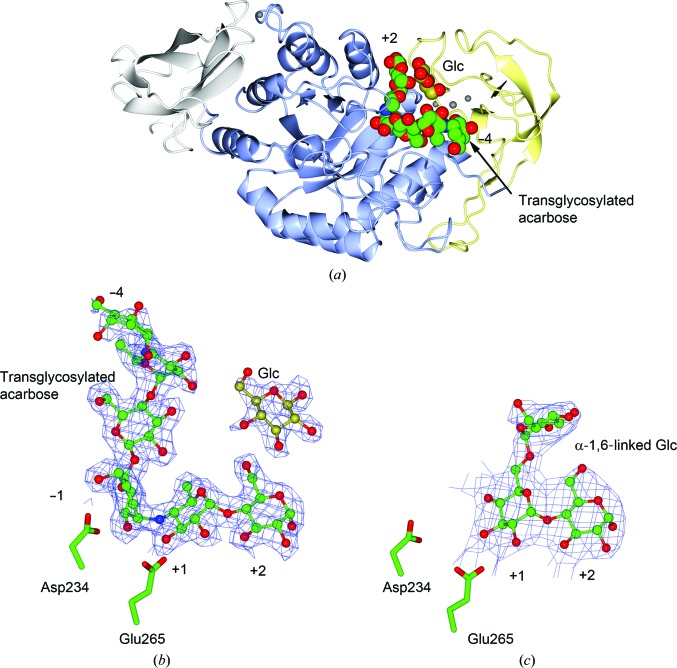
Three-dimensional structure of the *Alicyclobacillus* α-amylase AliC. (*a*) Three-dimensional protein cartoon, coloured by domain, with metal ions shown as shaded spheres and the acarbose and the +2′ glucose shown as van der Waals spheres. (*b*) Electron density for the transglycosylated acarbose in subsites −4 to +2 (and +2′ Glc) binding; density for an isolated ‘+3’ glucose is not shown. (*c*) Electron density for the binding of the branched oligosaccharide in subsites +1, +1 and +1′. Electron-density maps are *REFMAC* maximum-likelihood-weighted 2*F*_o_ − *F*_c_ syntheses contoured at 1σ. This figure was drawn with *CCP*4*mg* (McNicholas *et al.*, 2011 ▸).

**Figure 2:**
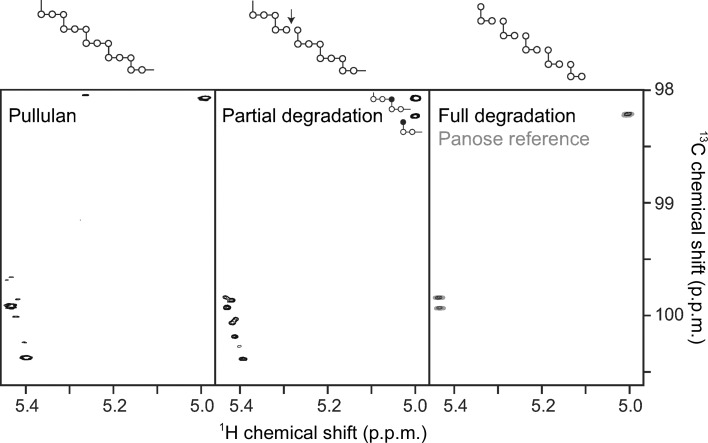
HSQC spectra of pullulan degradation by purified *Alicyclobacillus* α-amylase recorded with extensive sampling of the ^13^C dimension. Only the α-anomeric spectral region is shown. Three different glucopyranosyl units occur in pullulan (left). Signals of α-1,6 anomeric glucopyranosyl units at the nonreducing end emerge (see the inset in the middle spectrum) owing to cleavage at the indicated position (middle top). Pullulan is degraded to panose as the final product (right), as demonstrated by comparison with an authentic standard (grey). These experiments identify the pullulan-cleaving activity of *Alicyclobacillus* α-amylase as that of a panose-forming neopullulanase (EC 3.2.1.135).

Of particular interest to us was the observation of a ‘lone’ ordered glucose moiety that was not covalently linked to the acarbose-derived oligosaccharide in a position that could be indicative of the accommodation of branch points at either the +2 or +3 positions of AliC. An additional isolated glucose molecule, modelled in both α and β anomeric forms (occupancy set at 0.5 for each and in different orientations; omitted from Fig. 1 ▸ for clarity), was observed near a potential +3 site. We speculated whether these isolated glucose moieties could provide insight into branch-point recognition. The accommodation of branch points was therefore investigated by an analysis of limit digestion products (the characteristic oligosaccharides remaining after enzymatic digestion) on both pullulan (a regular linear polysaccharide of α-1,4, α-1,4, α-1,6 repeating trisaccharides) and amylopectin, an α-1,6-branched starch structure. The action of AliC on pullulan results in the production of the trisaccharide panose, glucose α-1,6-glucose α-1,4-glucose (Fig. 2 ▸), demonstrating that the enzyme must be able to accommodate α-1,6 linkages to glucose moieties in both the +1 and −2 subsites.

**Figure 3:**
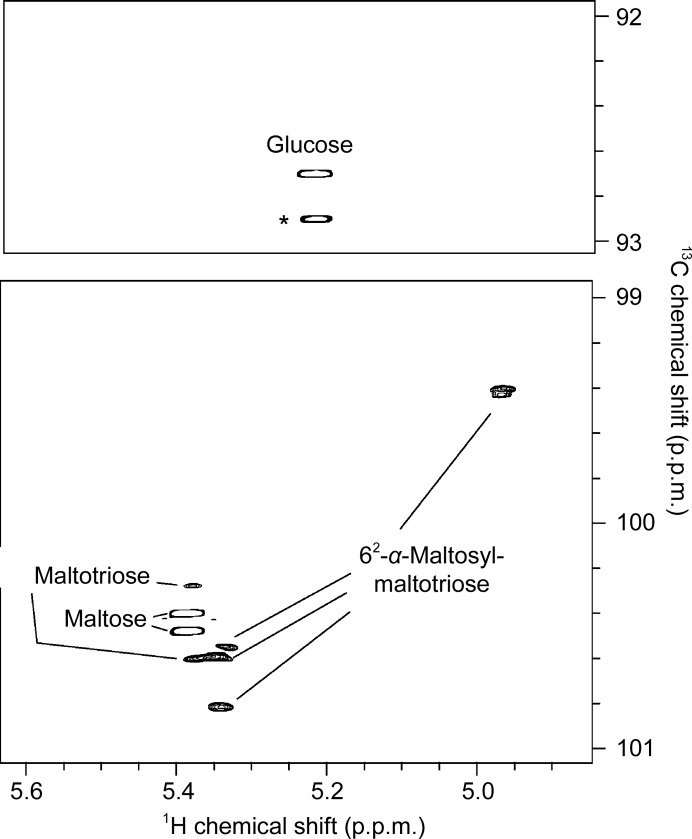
^1^H–^13^C HSQC NMR spectrum of degradation products from potato amylopectin using purified *Alicyclobacillus* α-amylase. Glucose, maltose, maltotriose and 6^2^-α-maltosylmaltotriose are the main products. The asterisk denotes the overlapping reducing-end α-glucopyranosyl signal of oligosaccharides. Several signals are detected for each molecule, as the individual glucopyranosyl units in oligosaccharides give separate signals.

**Figure 4:**
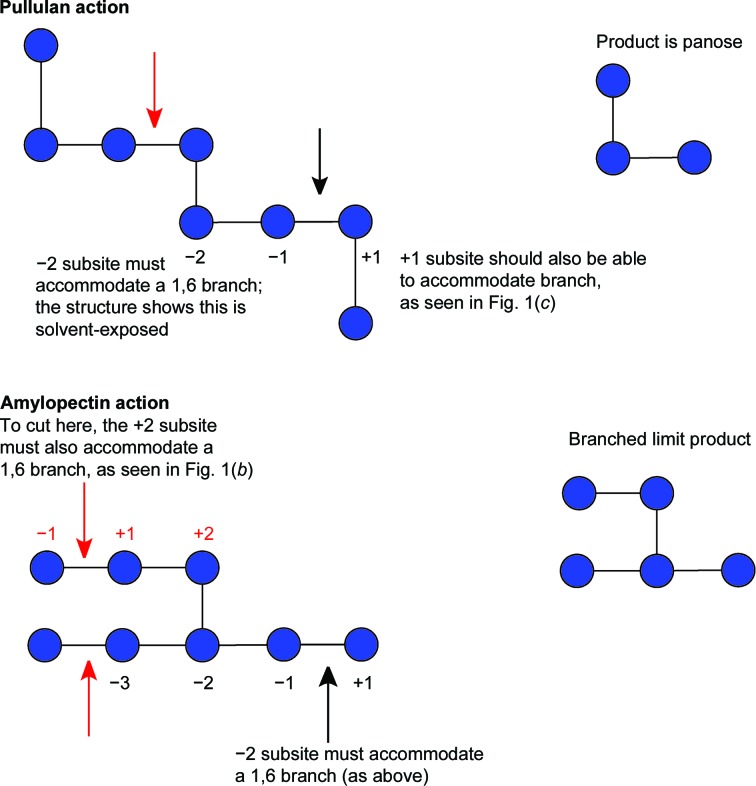
Interpretation of the limit digest patterns in terms of protein structure. Black and red arrows indicate the two/three places at which bonds must be cut to accommodate the limit dextrins observed. To generate panose from pullulan, both the +1 and −2 subsites must accommodate α-1,6 branches. The branched-ligand complex shows how subsite +1 can accommodate a branch (Fig. 1*c*), and in subsite −2 the O6 of acarbose was solvent-exposed. To generate the branched limit dextrin from amylopectin (Fig. 3 ▸), AliC must also be able to accommodate branching in the +2 subsite, which is consistent with the glucose moiety seen adjacent to O6 of the +2 sugar (Fig. 1 ▸ *b*).

Action on amylopectin produced the limit dextrin 6^2^-α-maltosyl maltotriose (for NMR assignments, see Petersen *et al.*, 2015 ▸; Jodelet *et al.*, 1998 ▸) (Fig. 3 ▸) demonstrating that AliC must also be able to accommodate starches with α-1,6 branches in both the −2 and +2 subsites. Taken together, the action on pullulan and amylopectin shows that AliC is able to accommodate 1,6 linkages in the −2, +1 and +2 subsites (Fig. 4 ▸).

### 3.3. Branched-ligand complex of AliC   

These branch patterns are consistent with the initial three-dimensional structure of AliC with the acarbose-derived oligosaccharide in which the −2 subsite O6 points into solvent and in which we observed a glucosyl moiety approximately where an O6 branch in either the +2 or +3 site might lie. In order to try and access a branched complex with a branch in the +1 subsite (the position of which is harder to model from the 6-deoxy sugars present in acarbose alone) we sought to obtain a complex by co-crystallizing AliC with 6^3^-α-d-glucosyl maltotriose (Megazyme, Wicklow, Ireland) and observing what was obtained with this active enzyme.

A ‘branched-ligand’ AliC complex was obtained through co-crystallization, with crystals forming in a new space group. This form diffracted poorly and data could only be obtained to 2.95 Å resolution. Weak density in the −1 subsite, largely diffuse but greater than would be expected for discrete solvent, remained unmodelled. Density was clearer for a panose tri­saccharide with an α-1,4-linked disaccharide in subsites +1 and +2 and, crucially, clear density for an α-1,6 branch accommodated in the +1 subsite (Fig. 1 ▸ *c*), providing a structural context for the limit digest analysis of action on amylopectin starch (Fig. 4). Notably, this +1′ sugar overlaps in position with the very recently reported SusG amylase branched-ligand complex (Arnal *et al.*, 2018 ▸).

## 4. Conclusions   

The accommodation of branch points in industrial enzymes is a key factor when considering their utility. How close to branch points an α-1,4 cleaving α-amylase will cleave defines what the ultimate limit dextrin product will be. The product profile impacts both on the cocktail of enzymes that are required for complete hydrolysis to glucose and on the physical properties of the limit dextrin itself (which are important in food and brewing processes, including the ‘mouthfeel’ of beer), such that insight into branch-point accommodation can provide powerful insight to aid protein-engineering campaigns. Yet, surprisingly, there has been very little structural insight into possible branch-point accommodation in α-amylases. Here, we have shown how serendipitous observation of a ‘lone’ glucosyl moiety close to the O6 position of an oligosaccharide complex inspired analysis of limit dextrins on substrates containing α-1,6 linkages, both linear and branched. Such combined X-ray and product-analysis NMR approaches should prove valuable in the future for interrogating, defining and ultimately exploiting the branch-point accommodation in this massively widespread family of starch-degrading catalysts.

## Supplementary Material

PDB reference: AliC, acarbose complex, 6gxv

PDB reference: branched-ligand complex, 6gya

## Figures and Tables

**Table 1:** Crystallization

	Acarbose complex	Branched-ligand complex
Method	Vapour diffusion, sitting drop	Vapour diffusion, hanging drop
Plate type	Swissci 96-well	Linbro 24-well
Temperature (K)	293	293
Protein concentration (mg ml^−1^)	20	20
Buffer composition of protein solution	25 m*M* borate, 2 m*M* CaCl_2_ pH 8 + 40 m*M* acarbose	25 m*M* borate, 2 m*M* CaCl_2_ pH 8 + 20 m*M* GMT
Composition of reservoir solution	0.2 *M* sodium citrate, 0.1 *M* BTP pH 6.5, 20% PEG 3350 (PACT G11)	20%(*w*/*v*) PEG 3350, 0.1 *M* BTP pH 8.5, 0.2%(*w*/*v*) sodium sulfate
Volume and ratio of drop	300 nl total, 1:1 ratio	1 µl total, 1:1 ratio
Volume of reservoir (µl)	54	1000

**Table 2:** Data collection and processing Values in parentheses are for the outer shell.

	Acarbose complex	Branched-ligand complex
Diffraction source	I04, DLS	I04, DLS
Wavelength (Å)	0.98	0.98
Temperature (K)	100	100
Detector	ADSC Quantum 315	PILATUS 6M Prosport+
Crystal-to-detector distance (mm)	254.8	475.8
Rotation range per image (°)	0.5	0.2
Total rotation range (°)	180	180
Exposure time per image (s)	0.5	0.2
Space group	*P*4_1_2_1_2	*P*6_1_
*a*, *b*, *c* (Å)	180.9, 180.9, 77.85	212.18, 212.18, 172.22
α, β, γ (°)	90, 90, 90	90, 90, 120
Mosaicity (°)	0.20	0.13
Resolution range (Å)	57.21–2.07 (2.12–2.07)	66.9–2.95 (3.03–2.95)
Total No. of reflections	1147294 (85223)	921282 (71482)
No. of unique reflections	78951 (5774)	92496 (6812)
Completeness (%)	99.6 (100)	100 (100)
Multiplicity	14.5 (14.8)	10.0 (10.5)
CC_1/2_ †	1.0 (0.907)	0.998 (0.914)
〈*I*/σ(*I*)〉	16.7 (4.5)	13.4 (2.7)
*R*_merge_ (%)	17.3 (74.5)	12.6 (74.8)
*R*_r.i.m._ ‡ (%)	18.4 (79.9)	14.1 (83.1)
Overall *B* factor from Wilson plot (Å^2^)	15.4	50.9

† CC_1/2_ values for *I*_mean_ are calculated by splitting the data randomly into half data sets.

‡ Estimated *R*_r.i.m._ = *R*_merge_ [*N*/(*N* − 1)]^1/2^, where *N* is the data multiplicity and *R*_merge_ is defined as 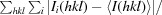 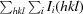, where *I*(*hkl*) is the intensity of the reflection.

**Table 3:** Structure solution and refinement

	Acarbose complex	Branched-ligand complex
PDB code	6gxv	6gya
Resolution range (Å)	57.21–2.07	66.86–2.95
No. of reflections, working set	78885	92448
No. of reflections, test set	3965	4492
Final *R*_cryst_	0.138	0.156
Final *R*_free_	0.176	0.183
Cruickshank DPI	0.14	0.37
No. of non-H atoms		
Protein	7770	15151
Ion	8	16
Ligand	272	312
Water	1092	224
Total	9142	15703
R.m.s. deviations		
Bonds (Å)	0.012	0.016
Angles (°)	1.5	1.8
Average *B* factors† (Å^2^)		
Protein	18 (6)	61 (13)
Ion	18 (8)	60 (9)
Ligand	34 (16)	92 (26)
Water	33 (13)	48 (10)
Ramachandran plot		
Most favoured (%)	920 (96.03%)	1830 (95.51%)
Allowed (%)	33 (3.44%)	76 (3.97%)
Outliers (%)	5 (0.52%)	10 (0.52%)

† Values in parentheses are standard deviations.
